# Evolutionary rate of SARS-CoV-2 increases during zoonotic infection of farmed mink

**DOI:** 10.1093/ve/vead002

**Published:** 2023-01-10

**Authors:** Ashleigh F Porter, Damian F J Purcell, Benjamin P Howden, Sebastian Duchene

**Affiliations:** Department of Microbiology and Immunology, The Peter Doherty Institute for Infection and Immunity, The University of Melbourne, Parkville, VIC 3010, Australia; Department of Microbiology and Immunology, The Peter Doherty Institute for Infection and Immunity, The University of Melbourne, Parkville, VIC 3010, Australia; Department of Microbiology and Immunology, The Peter Doherty Institute for Infection and Immunity, The University of Melbourne, Parkville, VIC 3010, Australia; Microbiological Diagnostic Unit Public Health Laboratory, The Peter Doherty Institute for Infection and Immunity, The University of Melbourne, Parkville, VIC 3010, Australia; Department of Microbiology and Immunology, The Peter Doherty Institute for Infection and Immunity, The University of Melbourne, Parkville, VIC 3010, Australia

**Keywords:** SARS-CoV-2, spillover, mink, evolutionary rate, molecular clock, spike gene

## Abstract

To investigate genetic signatures of adaptation to the mink host, we characterised the evolutionary rate heterogeneity in mink-associated severe acute respiratory syndrome coronaviruses (SARS-CoV-2). In 2020, the first detected anthropozoonotic spillover event of SARS-CoV-2 occurred in mink farms throughout Europe and North America. Both spill-back of mink-associated lineages into the human population and the spread into the surrounding wildlife were reported, highlighting the potential formation of a zoonotic reservoir. Our findings suggest that the evolutionary rate of SARS-CoV-2 underwent an episodic increase upon introduction into the mink host before returning to the normal range observed in humans. Furthermore, SARS-CoV-2 lineages could have circulated in the mink population for a month before detection, and during this period, evolutionary rate estimates were between 3 × 10^–3^ and 1.05 × 10^–2^ (95 per cent HPD, with a mean rate of 6.59 × 10^–3^) a four- to thirteen-fold increase compared to that in humans. As there is evidence for unique mutational patterns within mink-associated lineages, we explored the emergence of four mink-specific Spike protein amino acid substitutions Y453F, S1147L, F486L, and Q314K. We found that mutation Y453F emerged early in multiple mink outbreaks and that mutations F486L and Q314K may co-occur. We suggest that SARS-CoV-2 undergoes a brief, but considerable, increase in evolutionary rate in response to greater selective pressures during species jumps, which may lead to the occurrence of mink-specific mutations. These findings emphasise the necessity of ongoing surveillance of zoonotic SARS-CoV-2 infections in the future.

## Introduction

Coronaviruses (CoV) are zoonotic viruses associated with mammals and avian hosts (Wertheim et al. 2013) that are known for easily jumping species barriers due to high mutation rates, a large ribonucleic acid (RNA) genome (Lai and Cavanagh 1997; Su et al. 2016), and interaction with multiple angiotensin-converting enzyme 2 (ACE2) receptors, which enable viral entry and infection (Graham and Baric 2010; Ge et al. 2013). In recent decades, there have been three major outbreaks of CoV in the human population, causing epidemics: Severe acute respiratory syndrome (SARS), Middle East respiratory syndrome (MERS), and Coronavirus disease (COVID-19). SARS-CoV and MERS-CoV are thought to have originated in bats before spreading to the human population through an intermediate host (Cui, Li, and Shi 2019). SARS-CoV-2 likely has zoonotic origins, hypothesised to have initially spread from the Huanan seafood market in Wuhan, China (Hui et al. 2020; Liu and Saif 2020, Wu et al. 2020). CoV that circulate in the Chinese horseshoe bats (*Rhinolophus affinis* and *Rhinolophus malayanus*) are the closest known relatives to SARS-CoV-2 (although with estimated divergence from SARS-CoV-2 between 1948 and 1982) (Boni et al. 2020; Zhou et al. 2020a, 2020b). The Malayan pangolin (*Manis javanica*) has been cautiously suggested to be an intermediate host (Lam et al. 2020; Xiao et al. 2020; Zhang, Wu, and Zhang 2020), with much debate (Frutos et al. 2020; Liu et al. 2020). Minks are one of the many animals (Table 1) that are susceptible to SARS-CoV-2 infection but potentially one of the few that can transmit the virus back to humans (Larsen and Paludan 2020; Oreshkova et al. 2020; Larsen et al. 2021; Oude Munnink et al. 2021).

**Table 1.** A brief overview of non-human animals susceptible to SARS-CoV-2 infection.

Common name (scientific name)	Reference
Mink (*Neovision vision*)	Larsen and Paludan (2020), Larsen et al. 2021, Oude Munnink et al. 2021, Oreshkova et al. 2020.
Rhesus macaques (*Macaca mulatta*)	Deng et al. 2020, Munster et al. 2020.
Cynomolgus macaques (*Macaca fascicularis*)	Rockx et al. 2020.
African green monkeys (*Chlorocebus aethiops*)	Woolsey et al. 2021.
Common marmosets (*Callithrix jacchus*)	Lu et al. 2020
Domestic cats (*Felis catus*)	Halfmann et al. 2020, Ruiz-Arrondo et al. 2021, Shi et al. 2020
Domestic ferrets (*Mustela putorius*)	Kim et al. 2020, Schlottau et al. 2020, Richard et al. 2020.
Golden Syrian hamsters (*Mesocricetus auratus*)	Chan et al. 2020, Haagmans et al. 2021, Sia et al. 2020
Domestic rabbits (*Oryctolagus cuniculus domesticus*)	Mykytyn et al. 2021
Deer mice (*Peromyscus spp.*)	Fagre et al. 2021, Griffin et al. 2021.
Domestic dogs (*Canis lupus familiaris*)	Shi et al. 2020, Sit et al. 2020
Malayan tiger (*Panthera tigris jacksoni*)	Wang et al. 2020, McAloose et al. 2020
African lion (*Panthera leo leo*)	Koeppel et al. 2022
Tree shrews (*Tupaia belangeri*)	Zhao et al. 2020
White-tailed deer (*Odocoileus virginianus*)	Kuchipudi et al. 2021, Roundy et al. 2022
Fruit bats (*Rousettus aegyptiacus*)	Shi et al. 2020, Schlottau et al. 2020

**Figure 1.**
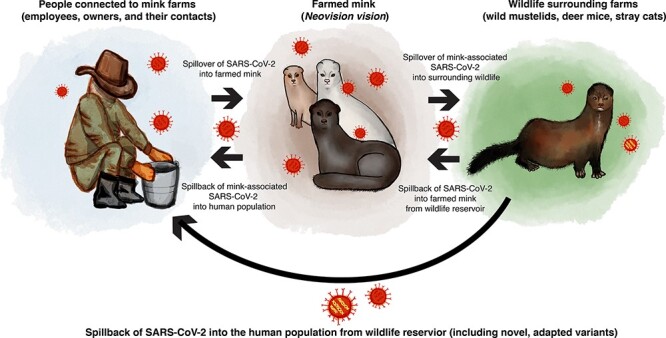
The illustration of the anthropozoonotic dynamics of mink-associated SARS-CoV-2, including human-to-mink, mink-to-mink, and mink-to-human transmission networks. To highlight the risk of viral reservoirs, we have shown a potential scenario of spill-back of mink-adapted lineages into the human population from infected wildlife.

In all zoonotic SARS-CoV-2 cases, human contact is likely the origin of transmission (Liu and Saif 2020). Although many sporadic spillover cases have occurred (Table 1), the first detected anthropozoonotic spillover event of SARS-CoV-2 occurred in mink farms (Fig. 1), with human-to-mink, mink-to-mink, and mink-to-human transmission networks established (Oude Munnink et al. 2021). The first report of SARS-CoV-2 in mink farms occurred late April 2020, in the Netherlands, followed by farms in Denmark during May, and in both countries, the outbreaks were sequenced comprehensively (Oreshkova et al. 2020; Hammer et al. 2021; Larsen et al. 2021; Lu et al. 2021). Further outbreaks were seen across Europe (Denmark, France, Poland, Lithuania, Latvia, Spain, Italy, Sweden, and Greece) and North America (the USA and Canada) (World Organisation for Animal Health 2022). All mink SARS-CoV-2 outbreaks originated from human infections (Oude Munnink et al. 2021), with multiple introductions of the virus into the mink population (Oreshkova et al. 2020), along with potential spread between farms (Oude Munnink et al. 2021). Mink-associated SARS-CoV-2 lineages form distinct clades, and the evolutionary rate of the virus is anticipated to increase in zoonotic transmission compared to human infections due to adaptive pressure upon introduction into a new host (Lu et al. 2021; Oude Munnink et al. 2021).

Mink-associated SARS-CoV-2 spread widely, both into the surrounding free-ranging mink (Aguiló-Gisbert et al. 2021; Shriner et al. 2021; Strang et al. 2022) and in ‘spill-back’ cases from infected minks into the human population (Oreshkova et al. 2020; Oude Munnink et al. 2021). Spill-back events present certain risks to public health. For example, spill-back of mink-associated lineages that have acquired mutations in the spike protein receptor–binding domain, which can lead to structural and/or functional changes in host receptor binding (Lan et al. 2020; Shang et al. 2020; Burkholz et al. 2021; Lu et al. 2021), presents a threat to human populations. This was observed when the mink-associated ‘Cluster 5’ lineage spread in Denmark. The ‘Cluster 5’ lineage had several mutations in the spike protein and spread widely in the human population surrounding the farms. Strikingly, the Cluster 5 lineage caused 40 per cent of the detected COVID-19 cases in the region, prompting governments to cull all farmed mink (Frutos and Devaux 2020; Larsen and Paludan 2020; Fenollar et al. 2021). The escape of mink-associated SARS-CoV-2 into the surrounding wildlife is also a major concern (Aguiló-Gisbert et al. 2021; Shriner et al. 2021; Strang et al. 2022), particularly as many farms border habitats that have high wild mustelid populations and other high-risk hosts (Shriner et al. 2021). An example of a high-risk host for a wildlife reservoir of SARS-CoV-2 is deer mice (*Peromyscus maniculatus*), which have no clinical symptoms but have high SARS-CoV-2 replication levels, enabling the formation of undetected viral reservoirs (Fagre et al. 2021). The establishment of a viral reservoir creates major issues for pathogen control and management, which has been observed previously in the case of the rabies virus forming a reservoir in wild-living raccoons and skunks (Rupprecht et al. 1995).

A recent debate concerning the origin of the first observed Omicron lineage (BA.1 and BA.2) has also emphasised the possibility of zoonotic spill-back events (Wei et al. 2021; Sun et al. 2022; Xu et al. 2022). Although the most popular theory for the emergence of Omicron is found in the increased mutational rate observed during the persistent infections in immunocompromised patients receiving antiviral therapy (Choi et al. 2020; Kemp et al. 2020), it has been suggested that mutations in Omicron were unlikely to have arisen during the evolution in the human host (Wei et al. 2021; Sun et al. 2022; Xu et al. 2022). Specifically, the forty-five point mutations unique to the Omicron lineage and distinct from the lineage’s nearest observed predecessor, lineage (B.1.1), have evolutionary signatures similar to mouse-adapted lineages. Most of these mutations cluster within the spike gene sequence, where many mutations overlap with mutations arising from chronic SARS-CoV-2 infection in mice (Wei et al. 2021) that increase the binding affinity to mouse ACE2 (Cameroni et al. 2021).

The spread of zoonotic SARS-CoV-2 to mink farms has highlighted the public health threat of spill-back events. The risk includes lineages that have undergone adaptation in farmed mink populations but also the spread and evolution of the virus in unmonitored reservoirs within wildlife populations (Larsen and Paludan 2020; Oude Munnink et al. 2021). Understanding how SARS-CoV-2 evolves when introduced into a new host is critical for managing these risks. Therefore, to explore the genetic signatures of adaptation to the mink host, we have estimated the evolutionary rate of mink-associated SARS-CoV-2 in comparison to the evolutionary rate observed within the broader SARS-CoV-2 phylogeny. We refer to the ‘evolutionary rate’ as the combination of substitutions and instantaneous mutations that are occurring in the genome over time, sometimes defined as the ‘evolutionary substitution rate’ (Ho et al. 2011). We utilised a range of molecular clock models that have been used to study the pattern of evolutionary rate variation during SARS-CoV-2 variant of concern (VOC) emergence and that of Ebola virus lineages (Mbala-Kingebeni et al. 2019, Tay et al. 2022). Based on the evidence that, during SARS-CoV-2 VOC emergence, there is an episodic increase in the evolutionary rate (Tay et al. 2022), we anticipate that a similar pattern will be observed in mink-associated SARS-CoV-2.

## Materials and methods

### Data collection

We downloaded a subset of SARS-CoV-2 isolates that were collected from minks (n = 69), along with a subset of the global human dataset (n = 200). To reduce the sampling bias in human isolates, we modified previous methods (Tay et al. 2022) and generated a representative global human dataset by randomly selecting 200 sequences from the Nextstrain SARS-CoV-2 build from April 2021 (Hadfield et al. 2018). We downloaded the sequences from the GISAID database (Elbe and Buckland-Merrett 2017; Shu and McCauley 2017) using GISAIDR (Wirth and Duchene 2022). We excluded low-coverage sequences, along with sequences generated from non-human hosts. We also removed all human isolates that had associations with potential zoonotic spill-back events from mink farms to focus exclusively on the period of host adaptation within mink populations.

For the mink isolates, we initially explored a total of 915 mink sequences from global farm outbreaks (Canada, the USA, Poland, Lithuania, Denmark, and the Netherlands). We generated an alignment in MAFFT v7 (Katoh et al. 2002; Katoh and Standley 2013) and a maximum likelihood phylogenetic tree using IQ-TREE2 (Minh et al. 2020). We utilised the phylogeny of mink-only isolates to identify large (more than thirty sequences), monophyletic clusters from a single location. We then repeated the alignment and maximum likelihood phylogeny with these sequences, along with the human isolates. From this tree, we only retained mink genomes that clustered in large monophyletic clades more than twenty sequences and >99 per cent bootstrap support) and that did not contain any human isolates (n = 69). Specifically, the mink sequences retained were sampled from two geographically distinct outbreaks, defined as the Netherlands clade (n = 29) and Denmark clade (n = 40). We used TempEst (Rambaut et al. 2016) to explore the temporal signal of the dataset before undergoing molecular clock model testing.

### Molecular clock models

We used a modified version of the previous methodology (Rambaut, McCrone, and Baele 2007; Mbala-Kingebeni et al. 2019; Tay et al. 2022); specifically, a range of Bayesian molecular clock models (Supplementary Table S1) were utilised to examine the patterns of the evolutionary rate variation between the mink and human clades (XML files are available at https://github.com/aporter704/Mink). The models range in describing the evolutionary rate along the specific branches within phylogenetic trees (Supplementary Fig. S1). We set up these models in BEAST 1.10 (Drummond and Rambaut 2007) using a Markov chain Monte Carlo of length 1 × 10^7^ and sampling every 10,000 steps. Each model was run in triplicate to verify the convergence to the same posterior distribution.

Of the models tested, the strict and relaxed (uncorrelated gamma distribution [UCG]) clock models range, respectively, from the simplest model (number of parameters, n = 1, the strict molecular clock) to the most complex (n = number of branches + 2, the relaxed uncorrelated molecular clock). The other models we applied, fixed local clock (FLC) models, enable evolutionary hypotheses as they require the definition of which branches will share an evolutionary rate a priori. This definition is usually based on a biological assumption, for example, a VOC lineage of SARS-CoV-2 to have a higher evolutionary rate than other lineages (Tay et al. 2022). We used six FLC models that have been described in detail previously (Tay et al. 2022). The first allows the evolutionary rate to vary within the mink clades, termed FLC (clade), or clades and along the stem, termed FLC (clade and stem), or only along the stem, termed FLC (stem). Additionally, these configurations were repeated where these rates could be shared with all mink clades, as in FLC (shared, stem), FLC (shared, clade), and FLC (shared, clade and stem). The biological theory behind models restricted to rate variability along the stem branches of mink clades is that the evolutionary rate is likely to increase over a short period of time during the adaptation to a new host and then returns to the background rate, as in FLC (stem). The rate could also vary along the stem and within the clade of a new host, such as the FLC (clade and stem) model, or only within the clade, as in FLC (clade). In all models, we included a priori knowledge that the time to the most recent common ancestor (tMRCA) of SARS-CoV-2 is estimated to be in the second half of 2019 (Duchene et al. 2020; Ghafari et al. 2022), with a prior distribution on the age of the root (Supplementary Table S2).

To assess the prior sensitivity of the FLC models, we specified a more informative rate prior for the mink-associated clades (Supplementary Table S2). We used a gamma distribution with shape = 1 and scale = 10^−2^ such that the 95 per cent percentile range was 2.5 × 10^–5^ to 3.7 × 10^–3^. This imposes a stronger penalty on high rates than the default prior in BEAST v.1.1.10 (Drummond and Rambaut 2007), which is known as the continuous time Markov chain (CTMC) reference prior. The CTMC prior is a gamma distribution with shape = 0.5 and rate = T, where T is tree length (sum of all branches in units of time) (Ferreira and Suchard 2008). This configuration was used to determine if the increased evolutionary rate observed in mink clades was not an artefact of the relatively wide distribution of the CTMC prior, as discussed in Tay et al. (2022). We also calculated Bayes factors to quantify the support for the rate change observed in the foreground branches with respect to the background. This form of Bayes factor consists of the ratio of the posterior and prior odds (Lemey et al. 2009) and differs from the standard application in phylogenetics where two models are compared via their marginal likelihoods (Oaks et al. 2019). The posterior odds are obtained by taking the proportion of Markov Chain Monte Carlo samples for which the foreground rate is higher than the background, resulting in a posterior probability, *P*, and dividing it by 1—P (i.e. posterior odds = *P*/1 − *P*). We conduct the equivalent procedure for the prior, and the ratio of posterior and prior odds, known as a Bayes factor, therefore, quantifies the amount of evidence in favour of a hypothesis given by the posterior (in this case that the foreground rate is higher than the background) relative to the prior.

### Mutation analysis

Within the mink-associated SARS-CoV-2 sequences included in our analysis, we identified mink-specific mutations, focusing on the spike protein gene. We identified four sites of interest, mutations Y453F, S1147L, F486L, and Q314K. To examine the evolution of these mutations within the broader SARS-CoV-2 phylogeny, we utilised an asymmetrical discrete trait analysis in BEAST v1.1.10 (Drummond and Rambaut 2007), using the amino acid site for each sequence as a state. We coded missing bases or deletions as an additional state. We used a similar model as the relaxed uncorrelated clock model with an underlying gamma distribution used earlier, with an exponential population size coalescent tree prior, a General Time Reversible substitution model with a gamma (+Γ) distrubution across sites, and a uniform distribution prior on the root age (Supplementary Table S2). We used a separate relaxed clock model for each site of interest but with the variance parameter fixed all partitions (i.e. all traits and the nucleotide sequence alignment).

## Results and discussion

### Distinguishing mink-associated clades within the SARS-CoV-2 phylogeny

**Figure 2.**
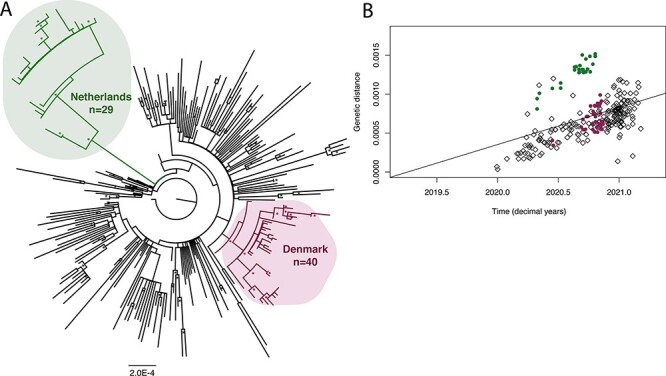
The phylogenetic analysis of mink-associated SARS-CoV-2 genomes, focusing on two geographical outbreaks from the Netherlands and Denmark. (A) Maximum likelihood tree of SARS-CoV-2 sequences (n = 269). The two mink-associated clades are highlighted in bubbles: the Netherlands clade (n = 29) is highlighted in the upper left corner and Denmark clade (n = 40) in the lower right corner, with the remaining tips representing human isolates (n = 200). The tree is rooted with SARS-CoV-2 reference sequence Wuhan/IVDC-HB-04/EPI_ISL_402120. Bootstrap replicates (n = 1000) >70 per cent are marked with an asterisk. The scale bar represents substitutions per site (subs/site). (B) A root-to-tip regression plot of the genetic distance vs time (in decimal years) of the 269 SARS-CoV-2 genomes used in this analysis. Sequences in the Netherlands group (n = 29) are represented by green dots, and Denmark sequences (n = 40) are represented by purple dots, with the remaining human isolates represented by black diamonds (n = 200).

We compiled a dataset of complete genomes with 269 taxa (n = 200 from human isolates and n = 69 from mink isolates; Supplementary Table S3), and the final alignment was 29,839 bp in length. We estimated a maximum likelihood tree (Fig. 2A), which revealed two distinct monophyletic mink-associated clades, as observed previously (Hammer et al. 2021). A pattern of increased genetic distance was observed in the Netherlands mink-associated SARS-CoV-2 sequences in a root-to-tip regression (Fig. 2B).

### An increased rate of evolution observed in stem branches

We used six molecular clock models in a Bayesian framework to explore the evolutionary rate heterogeneity within mink-associated SARS-CoV-2 clades (Table 2). The strict clock (SC) assumes that all branches have the same evolutionary rate and is thus a null model. The UCG clock assumes that branch rates were drawn from a gamma distribution and is the most liberal of all models compared here. The four FLC models represent hypotheses of branch rate variation and separate branch rates as belonging to two categories: ‘foreground’ and ‘background’ (Worobey, Han, and Rambaut 2014; Tay et al. 2022). Foreground branches are those assigned a different rate to the rest of the tree. The remaining branches in the tree, the background branches, represent the overall evolutionary dynamics of SARS-CoV-2 and serve as a comparison for the evolutionary rates estimated for the mink-associated clades, categorised as their geographical origin, the Netherlands and Denmark.

**Table 2.** Estimates generated from each molecular clock model. Estimates include tMRCA of the whole phylogeny, tMRCA of the Netherlands and Denmark clades, and the evolutionary rates (substitution/site/year) estimated for the whole phylogeny and the Netherlands and Denmark foreground evolutionary rates. The mean value is reported in each case and the 95 per cent HPD is shown in brackets.

Model	tMRCA (all)	tMRCA (Netherlands foreground)	tMRCA (Denmark foreground)	Estimated evolutionary rate	Estimated evolutionary rate (Netherlands foreground)	Estimated evolutionary rate (Denmark foreground)
SC	17 July 2019 [02 July 2019, 01 October 2019]	03 March 2020 [13 February 2020, 21 March 2020]	24 February 2020 [04 January 2020, 12 April 2020]	4.66 $ \times {10^{ - 4}}$ [4.25 $ \times {10^{ - 4}}$, 5.07 $ \times {10^{ - 4}}$]		
UCG	13 July 2019 [02 July 2019, 04 August 2019]	17 March 2020 [13 February 2020, 05 April 2020]	02 March 2020 [08 January 2020, 16 April 2020]	5.18 $ \times {10^{ - 4}}$ [4.64 $ \times {10^{ - 4}}$, 5.72 $ \times {10^{ - 4}}$]		
FLC (stem)	20 July 2019 [02 July 2019, 18 August 2019]	17 February 2020 [26 January 2020, 06 March 2020]^*^	17 February 2020 [28 December 2019, 08 April 2020]	4.82 $ \times {10^{ - 4}}$ [4.34 $ \times {10^{ - 4}}$, 5.2 $ \times {10^{ - 4}}$]^*^	1.2 $ \times {10^{ - 1}}$ [6.93$ \times {10^{ - 3}}$, 3.34 $ \times {10^{ - 1}}$]	4.42 $ \times {10^{ - 3}}$ [1.2 $ \times {10^{ - 5}}$, 2 $ \times {10^{ - 2}}$]
FLC (stem and clade)	17 July 2019 [02 July 2019, 17 September 2019]	14 March 2020 [24 February 2020, 28 March 2020]	31 December 2019 [10 November 2019, 13 February 2020]^*^	4.5 $ \times {10^{ - 4}}$ [4.08 $ \times {10^{ - 4}}$, 4.91$ \times {10^{ - 4}}$]^*^	1.86 $ \times {10^{ - 3}}$ [1.3 $ \times {10^{ - 3}}$, 2.45 $ \times {10^{ - 3}}$]	2.37$ \times {10^{ - 4}}$ [1.67 $ \times {10^{ - 4}}$, 3.1 $ \times {10^{ - 4}}$]
FLC (shared, stem)	24 July 2019 [02 July 2019, 22 August 2019]	17 February 2020 [30 January 2020, 06 March 2020]^*^	28 February 2020 [15 January 2020, 16 April 2020]	4.86 $ \times {10^{ - 4}}$ [4.45 $ \times {10^{ - 4}},$ 5.27 $ \times {10^{ - 4}}$]^*^	1.15 $ \times {10^{ - 1}}$ [6.1 $ \times {10^{ - 3}}$, 3.47 $ \times {10^{ - 1}}$]	
FLC (shared, stem and clade)	17 July 2019 [02 July 2019, 20 September 2019]	17 March 2020 [28 February 2020, 01 April 2020]	21 February 2020 [01 January 2020, 05 April 2020]	4.64 $ \times {10^{ - 4}}$ [4.25 $ \times {10^{ - 4}}$, 5.04 $ \times {10^{ - 4}}$]	1.9$ \times {10^{ - 3}}$ [1.33 $ \times {10^{ - 3}}$, 2.5 $ \times {10^{ - 3}}$]	

* Effective Sample Size (ESS) < 200.

The FLC stem model considers ‘foreground branches’ as those along the stems leading up to mink lineages (either the Netherlands or Denmark) and is consistent with an episodic change in the evolutionary rate (visualised in Fig. S1). In the FLC clade and stem model, the foreground branches include both the stem branch leading up to mink lineages and all branches within each independent mink clades, such that any changes in the evolutionary rate are maintained in the mink population (Fig. S1). We also specified alternative parameterisation of these two models but where the rate is shared amongst all mink foreground branches, as in FLC (shared, stem) and FLC (shared, clade and stem).

**Figure 3.**
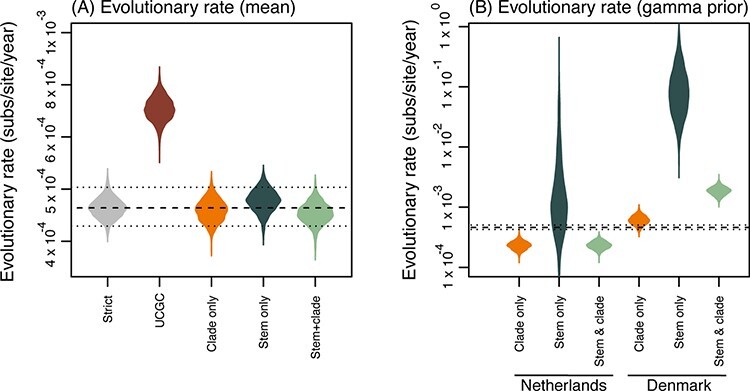
Violin plots of posterior density for the evolutionary rates (substitutions/site/year). (A) The mean evolutionary rates of each clock model, from left to right: strict clock, uncorrelated gamma distributed (UCGD) relaxed clock, and FLC models: clade only, stem only, and stem and clade. (B) Evolutionary rates estimated from FLC models using a conservative prior on clock rate. From left to right, estimates are shown for the foreground branches for the Netherlands, Denmark, and shared mink models: clade only, stem only, and stem and clade. Dashed lines represent the mean evolutionary rate (and 95 per cent HPD intervals) from the strict clock.

The mean evolutionary rates for the strict, FLC (clade), FLC (stem), and FLC (clade and stem) and all shared FLC models are slower than early estimates of SARS-CoV-2 evolutionary rates (Table 2 and Fig. 3A), which have ranged between 7 × 10^–4^ and 1.1 × 10^–3^ substitutions/site/year (Duchene et al. 2020; Ghafari et al. 2022) although our estimates are still within the uncertainty of previous estimates. For the mink-associated foreground branches, there was a much faster rate of evolution estimated for the FLC models that focused on the stem branch (Table 2, Supplementary Fig. S2), albeit with uncertainties that spanned several orders of magnitude, particularly for the Netherlands clade.

The stem-only estimates for the mink-associated foreground branches were considerably higher in the FLC (stem) model, with 12 × 10^–2^ (95 per cent highest posterior density [HPD]: 6.93 × 10^–3^, 3.34 × 10^–1^) and 4.42 × 10^–3^ (95 per cent HPD: 1.2 × 10^–5^, 2 × 10^–2^) for the Netherlands and Denmark foreground branches, respectively. Furthermore, this phenomenon was observed in estimates for FLC (shared, stem), with the shared mink foreground branches having an evolutionary rate of 11.5 × 10^–2^ (95 per cent HPD: 6.1 × 10^–3^, 3.47 × 10^–1^).

This pattern is also observed to a lesser extent in FLC (stem and clade) estimates for the Netherlands; however, the Denmark FLC (stem and clade) evolutionary rate was slower than the SC mean rate (Supplementary Fig. S2A). The evolutionary rate observed in the mink foreground branches in FLC (stem and clade, shared) appeared slightly faster than mean evolutionary rates (Supplementary Fig. S2B).

We also ran FLC models for independent and shared clades, FLC (clade) and FLC (clade, shared) (Supplementary Table S4) where estimates for the evolutionary rate within mink clades appear to have either a similar or slightly slower evolutionary rates when compared to the mean evolutionary rates (Supplementary Fig. S2).

We note that there appeared to be a slight increase in the evolutionary rate within the Netherlands foreground branches when compared to the Denmark foreground branches, as observed in all FLC models, along with the initial root-to-tip regression (Fig. 1B, Table 2, Supplementary Fig. S2).

The mean evolutionary rate of SARS-CoV-2 estimated from the UCG model, 5.18 × 10^–4^ (95 per cent HPD: 4.64 × 10^–4^, 5.72 × 10^–4^), was closer to previous estimates (Duchene et al. 2020; Ghafari et al. 2022) (Table 2). In a similar pattern to the FLC models, the stem branch leading to the Netherlands and Denmark clades within the summarised maximum clade credibility phylogeny had increased evolutionary rates of 1 × 10^–3^ (95 per cent HPD: 1 × 10^–4^, 2 × 10^–3^) and 8 × 10^–4^ (95 per cent HPD: 1 × 10^–4^, 1.5 × 10^–3^), respectively (Supplementary Fig. S3).

### Uncertainty in FLC (stems) model estimates

To explore the uncertainty observed in the mink-associated evolutionary rate estimates in the FLC models focusing on the stem branch, we conducted prior sensitivity analysis on the clock rates in all FLC models, which aligns with the recent estimates of the evolutionary rate of the virus (Ghafari et al. 2022) and penalises high evolutionary rate values (Supplementary Table S2). In particular, we specified the prior as a gamma distribution parameterised such that 95 per cent of the density lies between 2.5 × 10^–5^ and 3.7 × 10^–3^ substitutions/site/year. We distinguished these FLC models as FLC (stem*), FLC (clade*), FLC (stem and clade*), FLC (shared, stem*), FLC (shared, clade*), and FLC (shared, stem and clade*) (Table 3, Fig. 3B, Supplementary Table S5). There was still an observable increase in the evolutionary rate in mink-associated foreground branches in FLC (stem*) and FLC (shared, stem*) although to a lesser degree than the initial estimates (Table 3 and Fig. 3B).

**Table 3.** Estimates generated from local clock (FLC) models with a gamma prior on the clock rate.Estimates include the evolutionary rates (substitution/site/year) estimated for the whole phylogeny, and the Netherlands and Denmark foreground branches. The 95 per cent HPD interval is shown in brackets.

Model	Estimated evolutionary rate (mean)	Netherlands evolutionary rate	Denmark evolutionary rate
FLC (stem*)	4.54 × 10^−4^ [4.13 × 10^−4^, 4.93 × 10^−4^]	1.83 × 10^−3^ [1.3 × 10^−3^, 2.41 × 10^−3^]	2.43 × 10^−4^ [1.76 × 10^−4^, 3.17 × 10^−4^]
FLC (shared, stem*)	4.78 × 10^−4^ [4.36 × 10^−4^, 5.2 × 10^−4^]	6.59 × 10^−3^ [3 × 10^−3^, 1.05 × 10^−2^]	

### Statistical support for each model

In all shared models, the Bayes factor in support of a rate increase (which is a calculation of posterior odds divided by prior odds, as a measure of statistical support in favour of hypothesis, Table 4) was of >17, such that there is seventeen times more evidence of an increase in the evolutionary rate in the posterior with respect to the prior (Oaks et al. 2019). Although in all models assessed, the Bayes factor in support of a rate increase for the Netherlands foreground branches was >10, this was not the case for the Denmark foreground branches (Table 4).

**Table 4.** Bayes factor for each molecular clock model used in this study.

	Bayes factor	
Model	The Netherlands	Denmark
FLC (stem)	∞	4.6
FLC (stem and clade)	∞	1
FLC (clade)	10.5	1
FLC (stem*)	∞	3.2
FLC (stem and clade*)	∞	1
FLC (clade*)	10.6	1
FLC (shared, stem)	∞	
FLC (shared, stem and clade)	∞	
FLC (shared, clade)	17.8	
FLC (shared, stem*)	∞	
FLC (shared, stem and clade*)	∞	
FLC (shared, clade*)	17.6	

Note that infinity values for the Bayes factor occur when all the posterior samples support an increase in the rate for foreground branches relative to the background.

### Divergence of mink-associated clades

In all models, the tMRCA for the whole phylogeny is approximately mid-2019, which was the oldest boundary set by our priors (Supplementary Table S2). The Netherlands clade tMRCA estimates ranged from the last days of 2019 until mid-March 2020, and the Denmark clades follow a similar pattern.

### The emergence of mutations Y453F, S1147L, Q314K, and F488L within mink-associated SARS-CoV-2

Within the SARS-CoV-2 nucleotide sequence alignment utilised in our analysis, we identified several mink-specific mutations within the spike protein gene. Within the Danish mink-associated SARS-CoV-2 sequences, we observed previously characterised deletion in the N-terminus, at nucleotide positions 203–209, leading to the loss of His and Val amino acids (respectively, H69∆ and V70∆) (Oude Munnink et al. 2021). Furthermore, we observed mutation Y453F in the receptor-binding domain (substitution of A to T at nucleotide position 1358, leading to an amino acid replacement of Tyr to Phe) (Devaux et al. 2021; Hoffmann et al. 2021; Oude Munnink et al. 2021), which has been shown to increase interactions with mink ACE2 (Tan et al. 2022), and potentially allows the evasion of neutralising antibodies in human infections (Hoffmann et al. 2021; Zhang et al. 2021). Additionally, we identified nonsynonymous mutation in the S2 subunit, at position 3440 (C to T), leading to a shift from Ser to Leu, termed S1147L (Devaux et al. 2021; Hoffmann et al. 2021; Oude Munnink et al. 2021). The final mutation identified, which is not widely discussed in the literature, is a synonymous mutation at nucleotide position 558 (C to T).

Within the Dutch mink-associated SARS-CoV-2 sequences, we observed previously characterised Q314K (substitution of C to A at position 940, leading to an amino acid substitution from Gln to Lys) and F486L (T to C mutation at position 1456, leading to a Phe to Leu amino acid substitution) (Burkholz et al. 2021). F486L has been observed in the receptor-binding motif in the spike protein gene of both SARS-CoV-1 and SARS-CoV-2 and in a range of animal samples including bats (*R. affinis*) in Yunnan, China, pangolins (*M. javanica*), and mink (Burkholz et al. 2021). F486L variants also display a decreased sensitivity to neutralising monoclonal antibodies in humans (Hayashi, Yaegashi, and Konishi 2021). We also observed an insertion at position 429–43 (leading to an addition of Tyr, termed ins144), a substitution at 784 (G to T) causing a change from Ala to Ser (262) termed A262S, and a synonymous mutation of A to G at position 3300.

**Figure 4.**
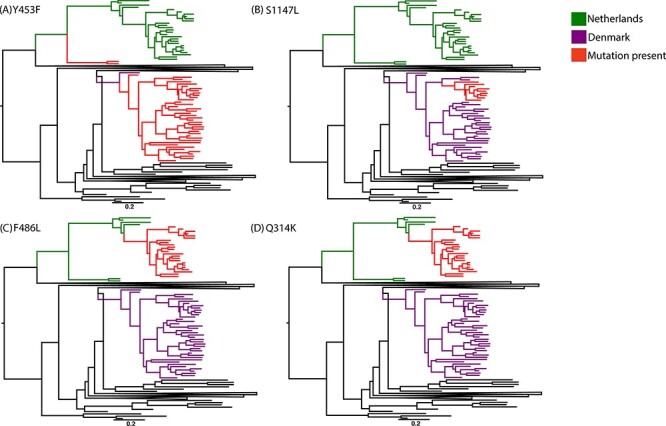
Maximum clade credibility phylogenies highlighting the two mink-associated SARS-CoV-2 clades. Mink-associated clades are shown in green and purple, for outbreaks in the Netherlands and Denmark, respectively, and for each site of interest, tips with the mutation present are shown in red. Mutations (A) Y453F, (B) S1147L, (C) F486L, and (D) Q314K are visualised. Each tree is mid-point rooted for clarity, and a scale bar represents substitutions/site/year.

To examine the mink-specific mutations, we selected four sites of interest: mutations Y453F, S1147L, F486L, and Q314K, and examined the rate at which these amino acid substitutions occur per site per year (Fig. 4).

Mutation Y453F occurs in both the Denmark and Netherlands outbreaks (Fig. 4A). However, in the Netherlands clade, it is only present in two sequences that are distinct from the rest of the clade. The respective samples were from an early detection of the mink outbreak, collected on 29 April 2020. The rest of the Dutch samples were collected from May–October. In the Danish clade, Y453F was present in all sequences except for one, which was one of the earliest collected samples. In both clades, the rate of amino acid change (in units of amino acid substitutions/site/year) was slightly higher along the branch most likely to be the ‘mutation zone’, which we assume to be both the temporal space where the mutation occurred and ancestor of future sequences that contain the mutation. The rate in the mutation zones of Y453F was 0.05 (95 per cent HPD: 0.04, 0.12) compared to the mean rate (0.04, 95 per cent HPD: 0.01, 0.06).

Mutation S1147L is present only in the Danish outbreak (Fig. 4B). S1147L was present in eight sequences, which were sampled from 7 October to 9 November 2020. The rate in the S1147L mutation zone was also slightly increased compared to the mean, respectively, 2 × 10^–2^ (95 per cent HPD: 2 × 10^–4^, 4 × 10^–2^) to 1 × 10^–2^ (95 per cent HPD: 3.9 × 10^–4^, 3 × 10^–2^).

Both mutations F486L and Q314K were present only in the Dutch outbreak and in the same twenty-three sequences (sampled 20 August to 21 October). The rate in both mutation zones was increased in comparison to the mean rate. The mutation zone in Q314K had a rate of 0.09 (95 per cent HPD: 0.1, 0.21) compared to mean rate 0.07 (95 per cent HPD: 0.03, 0.1). The mutation zone in F486L had a rate of 0.11 (95 per cent HPD: 0.08, 0.25) compared to mean rate of 0.03 (95 per cent HPD: 0.01, 0.06).

### Positive selection during host adaptation in SARS-CoV-2

First, we note that the evolutionary rates observed in Table 2 are slower than the early reports of the SARS-CoV-2 evolutionary rate, which range from 7 × 10^–4^ to 1.1 × 10^–3^ (Duchene et al. 2020; Ghafari et al. 2022). However, the long-term substitution rate presented by Ghafari et al. (2022) is only slightly slower than our estimates, suggesting that these estimates align with the long-term evolutionary rates of SARS-CoV-2.

It was anticipated that upon introduction into the mink host, SARS-CoV-2 would undergo adaptive evolution, as seen previously in SARS-CoV during the adaptation to the human host after jumping from palm civets (*Paradoxurus hermaphroditus*) (Chinese SARS Molecular Epidemiology Consortium 2004; Song et al. 2005). A substantial change in the evolutionary rate (an approximate nine-fold increase in mutations accumulated per month in comparison to previous estimates) was observed along the stem branch, leading to the mink-associated clades. We hypothesise that the observed change in the evolutionary rate along the stem branches leading to the mink clades is evidence of positive selection occurring during the strong selective adaptation of SARS-CoV-2 to the mink host. Importantly, this adaptive phase is episodic, as this pattern of increased evolution does not appear to continue within the mink clades when no longer crossing a species barrier (Fig. 3). A similar phenomenon was observed in SARS-CoV-2 VOCs, where positive selection was observed along the stem branches (particularly in the case of the Alpha lineage) but not within the VOC clades (Tay et al. 2022).

We suggest that this increased rate of evolution along the stem branch could lead to a dramatic shift in the mutations accumulated in the lineages circulating amongst the farmed mink populations. The estimates produced under the FLC (shared, stem) model (Fig. 3C) suggest the evolutionary rate was much more rapid in the mink clades, a trend that continued to be observed even with a more conservative prior in the FLC (shared, stem*) model (Fig. 3B), with estimates of the evolutionary rate averaging 6.59 × 10^–3^ substitutions/site/year (95 per cent HPD: 3 × 10^–3^, 1.05 × 10^–2^ Bayes factor = ∞; note that infinity occurs when the posterior density is fully concentrated on foreground branches having a higher rate than the background). Based on these estimates, the virus could accumulate approximately sixteen mutations per month (with a 95 per cent HPD of 7-26 mutations), which is a dramatic increase from the mean evolutionary rate of SARS-CoV-2 (approximately two mutations per month). However, we note that it is unexpected that the Denmark clade does not appear to have a strong signal for an increased evolutionary rate in comparison to the Netherlands clade (Table 4). There are many underlying factors within the mink farm outbreaks that could contribute to such a difference in results; for example, we do not know how many times SARS-CoV-2 spilled over into the mink population or for how long it was circulating in the population before detection. Furthermore, in both Dutch and Danish analyses, isolates from multiple farms were pooled, and within farms, there were differences in mink population, as well as the proportion of adult and kit populations. The transmission dynamics are likely to differ between farms, and furthermore, it is not understood if the duration of infection varies between kit and adult mink. We anticipate that these differences (and other environmental factors, such as temperature) influenced the evolutionary rate estimates for both outbreaks. We highlight this as a critical consideration for future spillover events.

### Signatures of animal adaptation in zoonotic SARS-CoV-2

The broad zoonotic potential (Table 1) and generalist nature of SARS-CoV-2 have been emphasised (Tan et al. 2022), with minimal adaptation required for zoonotic spillovers in novel hosts. In mink populations, identical mutations have arisen independently in the virus (Tan et al. 2022). In SARS-CoV-2 isolates from both mink and white-tailed deer populations, there have been six mutations predicted to be associated with animal adaptation (Tan et al. 2022) and twenty-three recurrent mutations (including three nonsynonymous mutations in the receptor-binding domain of the spike protein) have arisen at least four times in mink-associated SARS-CoV-2 but are rarely seen in human samples (Tan et al. 2022). This is a substantial number of mutations to have accumulated in such a short period, with previous estimates of the evolutionary rate of SARS-CoV-2 (Duchene et al. 2020) requiring a year to accumulate twenty-three mutations. Similarly, under previous evolutionary rate estimates, the eighteen mutations observed along the stem branch leading to the Netherlands mink clade would have taken approximately a year to accrue; however, when accounting for a rate increase along the stem branch, it is reduced to months, which is more accurate for the timeline of SARS-CoV-2 outbreaks in mink farms.

When examining the specific mutations that have arisen in mink-associated SARS-CoV-2, it appears that mutation S1147L appeared once the Danish outbreak had been established and was only found in nine sequences. Importantly, mutation S1147L has also appeared in Omicron variants circulating in the USA (Ou et al. 2022). We recommend the monitoring of lineages that contain this mutation as it might have significance for host interactions.

Interestingly, mutations F486L and Q314K appeared to be linked, as they appeared in the same twenty-three sequences within the Dutch outbreak. Future work is required to determine if this pattern is random or if these mutations coexist for a biological purpose.

Mutation Y543F arose early in both mink outbreaks (Oude Munnink et al. 2021) but only became established in Danish lineages (Fig. 4A). Mutation Y453F have been reported in humans and both American mink (*Neovision vision*) and European minks (*Mustela luterola*) (Devaux et al. 2021). Previous work has suggested that the accumulation of mutation Y453F has direct impacts on ACE2 interactions and that compensatory mutations may have arisen during interspecies transmission (Frutos et al. 2022). Furthermore, a SARS-CoV-2 sequence isolated from lymphoma patient with chronic COVID-19 contained Y453F, H69∆, and V70∆ mutations and did not appear to be related to the mink clusters, suggesting independent acquisition. Further evidence suggests that these mutations were accumulated as part of the host adaptation response, and these mutations were identified at intermediate frequencies in the patient, suggesting intra-host polymorphism (Bazykin et al. 2021). This, along with the frequency of these mutations in mink-associated SARS-CoV-2, indicates parallel evolution under two different forms of selection. This emphasises the importance of tracking the Y453F mutation in both human and animal SARS-CoV-2 infections.

We assume that both host-specific mutations and an increased rate of evolution would not be unique to the introduction of SARS-CoV-2 into the mink host and that this phenomenon may be seen in other novel hosts where inter-host transmission is possible. For example, an additional anthropozoonotic spillover event (and potential reservoir of concern) is the wild and captive white-tailed deer (*Odocoileus virginianus*) population in North America (Kuchipudi et al. 2021; Roundy et al. 2022). A third of deer tested in Iowa, and thirty-four out of thirty-six deer tested positive at a captive cervid facility in Texas were positive for SARS-CoV-2 RNA, with evidence of deer-to-deer transmission (Kuchipudi et al. 2021; Roundy et al. 2022; Willgert et al. 2022). Additionally, SARS-CoV-2 with several mutations has been detected in white-tailed deer in Québec, Canada (Kotwa et al. 2022). Phylogenetic and epidemiological analyses have linked a possible deer-to-human transmission event with a novel, highly divergent lineage of SARS-CoV-2 detected in white-tailed deer (Pickering et al. 2022). Although there is no direct evidence of deer-to-human transmission, a recent work has also highlighted the potential of spill-back events from infected deer populations (Willgert et al. 2022). Furthermore, the adaptation of SARS-CoV-2 to the rodent host, and subsequent spill-back into the human population, could explain the emergence of divergent Omicron lineage in the late 2021 (Wei et al. 2021; Sun et al. 2022; Xu et al. 2022). It has been suggested that, while adapting within a rodent host, SARS-CoV-2 accumulated mutations for approximately 12 months before re-entering the human population as the Omicron lineage (Wei et al. 2021) with preliminary evidence for increased infectivity (Chen et al. 2022) and higher levels of ‘vaccine-breakthrough’ (Andrews et al. 2021).

### Detection and surveillance of zoonotic SARS-CoV-2

Estimates of divergence for the Netherlands and Denmark clades suggest that they emerged in the first months of 2020 (Table 2). The outbreaks were first detected in the Netherlands and Denmark during late April and early May, respectively; however, our estimates suggest that SARS-CoV-2 was circulating in the mink population a month before detection, or more likely, the ancestral lineages that were present in the ‘stem branch’ period were undersampled. We note that it is likely that the lack of full diversity of mink-associated SARS-CoV-2 in our dataset means that the tMRCA estimated here is a lower bound. Furthermore, the tMRCAs for mink-associated SARS-CoV-2 may have a slight downward bias due to the overall tMRCA estimates, which pre-date other reported tMRCAs of SARS-CoV-2 by several months, although they are within the range of uncertainties (Rambaut 2020).

We acknowledge that the population structure and dynamics, along with respective sampling strategies, of human and mink datasets likely have differences that will impact and potentially bias our estimates of both the evolutionary rate and tMRCA. As discussed previously (Mavian et al. 2020; Porter et al. 2022), the sampling bias within the SARS-CoV-2 dataset can impact phylodynamic analysis, and future studies will benefit from the strategic sample collection from both human and non-human hosts during spillover events. Furthermore, in future outbreaks in animal populations, tactical sampling over both temporal and geographical ranges will assist future studies to estimate more informative evolutionary rates within non-human hosts (i.e. having a monophyletic clade of >100 animal-associated sequences). The use of structured tree prior would explicitly address such sampling bias, but we note that informative sequence datasets are usually robust to misspecification of the tree prior (Ritchie, Lo, and Ho 2017; Möller, du Plessis, and Stadler 2018).

Due to the magnitude of farmed mink populations (in both population size and geographical reach), in addition to the established transmission pathways (Fig. 1) and the ability of SARS-CoV-2 to accumulate potentially harmful mutations rapidly, zoonotic viral transmission poses a significant threat to global public health (Oreshkova et al. 2020; Sharun et al. 2021). Furthermore, the formation of a permanent reservoir of SARS-CoV-2 in wildlife populations could lead to spill-back events of animal-adapted lineages of the virus into the human population and other susceptible animals (Manes, Gollakner, and Capua 2020; Delahay et al. 2021; Kotwa et al. 2022; Pickering et al. 2022; Willgert et al. 2022). Our work emphasises the necessity of a ‘One Health’ approach to surveillance: to track any zoonotic spread of SARS-CoV-2, identify outbreaks in novel hosts rapidly, and monitor the ongoing spread of SARS-CoV-2 after host-switching to prevent the establishment of a viral reservoir. In particular, monitoring ‘at-risk’ animal groups is essential, including farmed and wild-living populations of minks and white-tailed deer, animals that regularly come into contact with humans (Wei et al. 2021; Zhou and Shi 2021), and species that host CoV closely related to SARS-CoV-2 (such as members of the genus *Rhinolophus*) (Delaune et al. 2021). This surveillance system relies greatly on whole genome sequencing, which has played a key role in monitoring the emergence and evolution of other variants (European Centre for Disease Prevention and Control 2020; Ou et al. 2022; Tay et al. 2022). In this study, we highlight the power of the extensive whole genome sequencing of SARS-CoV-2 isolates collected during the 2020 outbreaks in mink farms and recommend that this remains a high priority for future zoonotic spillover events of SARS-CoV-2. For the future, we encourage applying a One Health approach to zoonotic SARS-CoV-2 surveillance (Greenhorn et al. 2021), focusing on the intersections of ecological, animal, and public health. Lastly, we highlight that the continuation of sampling, sequencing, and sharing data is critical for our ability to monitor the evolutionary dynamics of zoonotic SARS-CoV-2.

## Supplementary Material

vead002_Supp

## Data Availability

All data are available in the main text or the supplementary materials.
