# Supplementary material for: Evolutionary rate of SARS-CoV-2 increases during zoonotic infection of farmed mink
Source: Virus Evol. 2023 Jan 10;9(1):vead002. doi: 10.1093/ve/vead002 (PMC9896948; doi:10.1093/ve/vead002)
Supplement: vead002_Supp [file vead002_supp.zip › suppl_data/Supplementary Materials.docx]

**Supplementary Materials**

Evolutionary rate of SARS-CoV-2 increases during zoonotic infection of farmed mink

Ashleigh F. Porter, Damian F.J. Purcell, Benjamin P. Howden, Sebastian Duchene

**Supplementary Figures S1-S3**

**Supplementary Tables S1-S5**


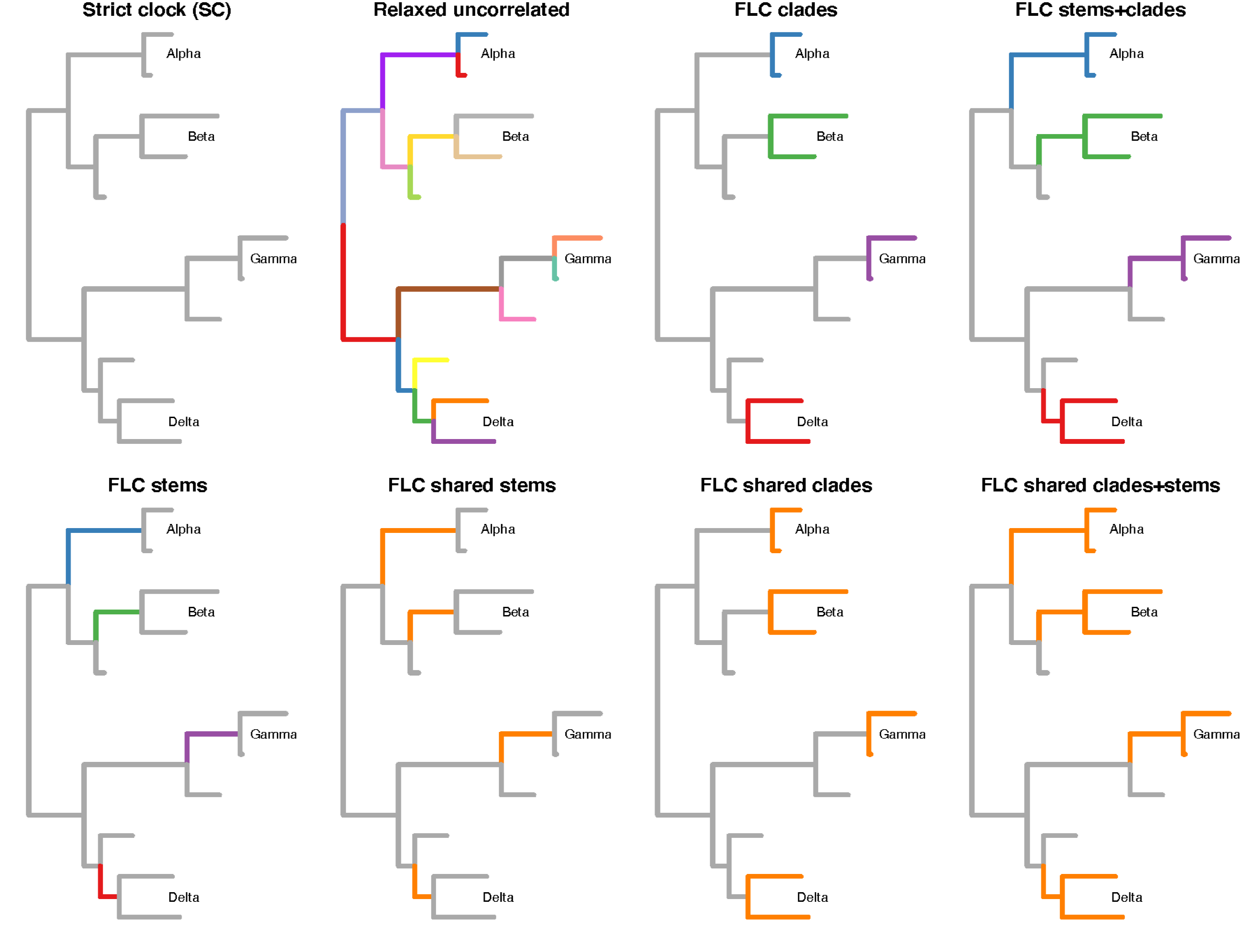


**Fig. S1**. **Visualization of the molecular clock model testing methodology.** Methods have been modified from Tay et al. (2022), with eight molecular clock models applied in hypothetical trees with 4 clades of interest (A, B, C and D). Figure adapted from Tay et al. 2022.


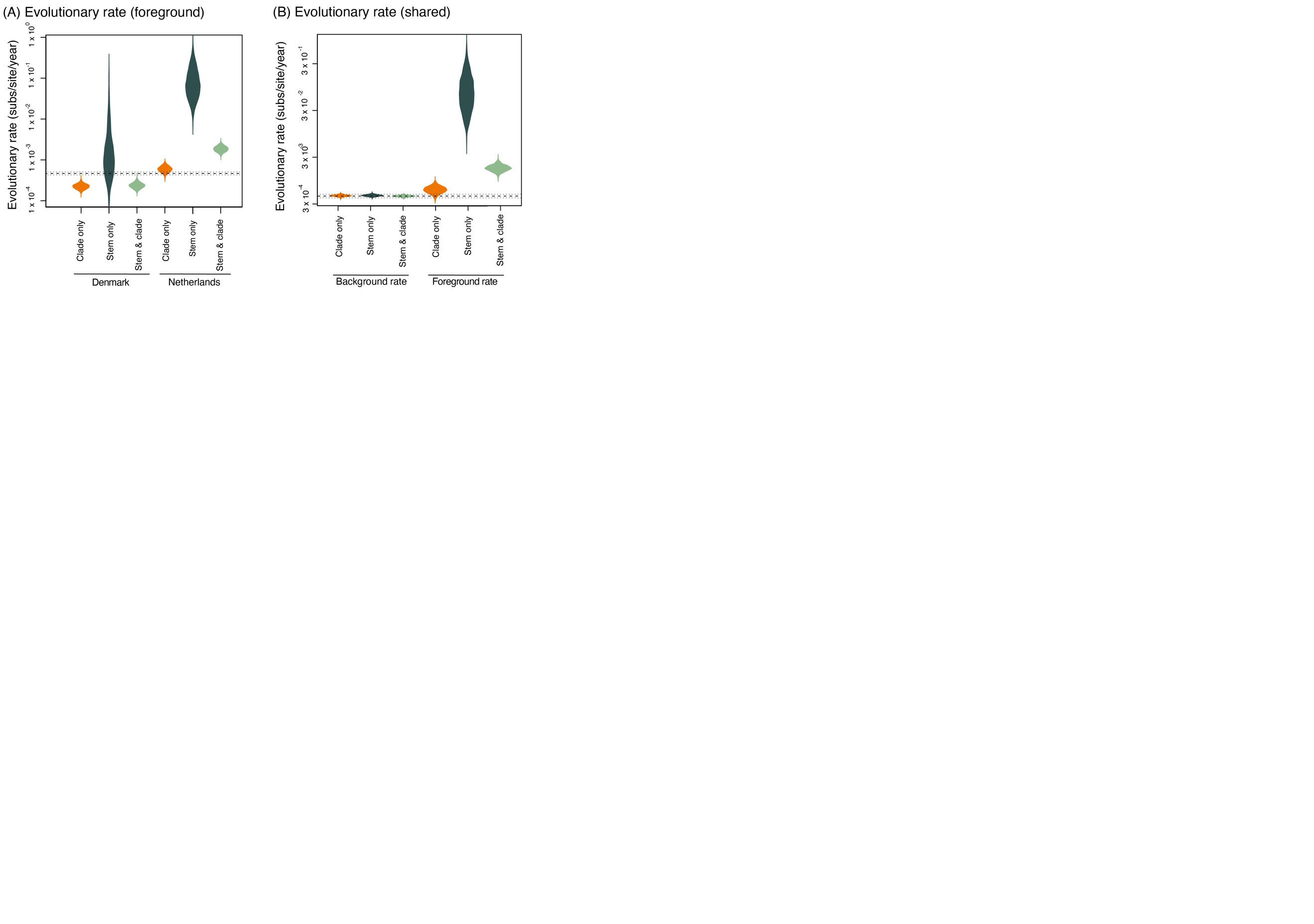


**Fig. S2**. **Violin plots of posterior density for the evolutionary rates (substitutions/site/year). (**A) Evolutionary rates estimated for the Netherlands and Denmark foreground branches using FLC models: clade only (orange), stem only (dark green) and stem and clade (light green). (B) Evolutionary rates estimated for the background and foreground branches of the shared mink FLC models: clade only (orange), stem only (dark green) and stem and clade (light green).


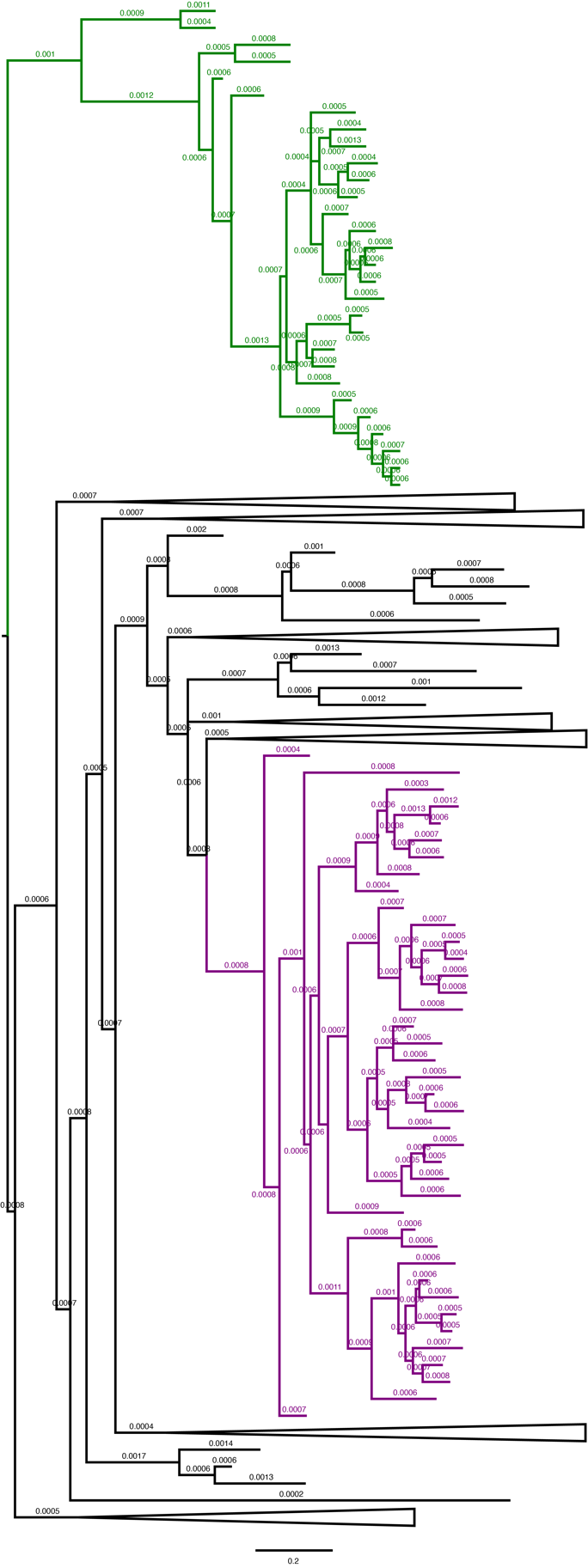


**Fig. S3**. **Maximum clade credibility tree of the UCGD model.** The Netherlands clade is highlighted in green, and the Denmark clade is highlighted in purple. Evolutionary rates are shown above each branch. The tree was mid-point rooted and several human-associated clades were collapsed for clarity. The scale bar below represents substitutions/site/year.

**Table S1. Molecular clock models used in model testing.**

| Clock model | Abbreviation | Parameters |
| --- | --- | --- |
| Strict clock | SC | Clock rate (*n=1)* |
| Uncorrelated gamma-distributed relaxed clock | UCGD | Total branch number, mean, sd *(n=269+1+1)* |
| Fixed local clock (clades) | FLC (clade) | Background rate, clade number (1+2) |
| Fixed local clock (stems) | FLC (stem) | Background rate, stem number (1+2) |
| Fixed local clock (clades & stems) | FLC (stem & clade) | Background rate, clade/stem number (1+2) |
| Fixed local clock (shared clades) | FLC (shared, clade) | Background rate, clade number (1+1) |
| Fixed local clock (shared stems) | FLC (shared, stem) | Background rate, stem number (1+1) |
| Fixed local clock (shared clades & stems) | FLC (shared, stem & clade) | Background rate, clade/stem number (1+1) |

**Table S2.** **Specific priors used in molecular clock models.**

| Models with prior | Parameters |
| --- | --- |
| All models | Uniform distribution on age(root) (2019.5, 2020). |
| All models | CTMC scale prior for the clock.rate (scaleFactor = 0.75, weight = 3). |
| FLC(stem*), FLC(clade*), FLC(stem & clade*), FLC(shared, stem*), FLC(shared, clade*), FLC(shared, stem & clade*) | Gamma distribution for clock.rate (shape =1, scale = 0.001) for the foreground branches only (Netherlands, Denmark or shared rate). |

**Table S3. SARS-CoV-2 sequences used in this study.**

| GISAID Accession ID |
| --- |
| EPI_ISL_1001001  EPI_ISL_1001002  EPI_ISL_849377  EPI_ISL_430818  EPI_ISL_856766  EPI_ISL_1139148  EPI_ISL_1139151  EPI_ISL_1013818  EPI_ISL_522686  EPI_ISL_678316  EPI_ISL_1121039  EPI_ISL_812423  EPI_ISL_854745  EPI_ISL_885139  EPI_ISL_806729  EPI_ISL_794718  EPI_ISL_667780  EPI_ISL_944741  EPI_ISL_654807  EPI_ISL_467989  EPI_ISL_516550  EPI_ISL_740874  EPI_ISL_767859  EPI_ISL_849747  EPI_ISL_854748  EPI_ISL_693297  EPI_ISL_812436  EPI_ISL_451613  EPI_ISL_530231  EPI_ISL_414414  EPI_ISL_755571  EPI_ISL_513911  EPI_ISL_544964  EPI_ISL_577607  EPI_ISL_592769  EPI_ISL_596712  EPI_ISL_854247  EPI_ISL_437942  EPI_ISL_636973  EPI_ISL_681308  EPI_ISL_483623  EPI_ISL_977658  EPI_ISL_722930  EPI_ISL_837553  EPI_ISL_955142  EPI_ISL_1016884  EPI_ISL_1181405  EPI_ISL_1240642  EPI_ISL_480298  EPI_ISL_918371  EPI_ISL_845548  EPI_ISL_1258234  EPI_ISL_1272402  EPI_ISL_1167761  EPI_ISL_746644  EPI_ISL_1168770  EPI_ISL_591278  EPI_ISL_402120  EPI_ISL_434534  EPI_ISL_1220065  EPI_ISL_956289  EPI_ISL_770029  EPI_ISL_1272023  EPI_ISL_1232316  EPI_ISL_1014556  EPI_ISL_817398  EPI_ISL_877555  EPI_ISL_481245  EPI_ISL_907112  EPI_ISL_1138530  EPI_ISL_995915  EPI_ISL_887175  EPI_ISL_788934  EPI_ISL_1197071  EPI_ISL_860184  EPI_ISL_1283938  EPI_ISL_862039  EPI_ISL_568689  EPI_ISL_582030  EPI_ISL_1254794  EPI_ISL_895035  EPI_ISL_686924  EPI_ISL_568746  EPI_ISL_1039223  EPI_ISL_968815  EPI_ISL_968827  EPI_ISL_1027645  EPI_ISL_1027652  EPI_ISL_1005230  EPI_ISL_904957  EPI_ISL_1191600  EPI_ISL_424667  EPI_ISL_1060683  EPI_ISL_979330  EPI_ISL_1040605  EPI_ISL_913984  EPI_ISL_1279462  EPI_ISL_516936  EPI_ISL_516931  EPI_ISL_768840  EPI_ISL_1159697  EPI_ISL_887472  EPI_ISL_964945  EPI_ISL_833041  EPI_ISL_461267  EPI_ISL_1210475  EPI_ISL_728656  EPI_ISL_579068  EPI_ISL_1016861  EPI_ISL_582022  EPI_ISL_794622  EPI_ISL_755622  EPI_ISL_843198  EPI_ISL_1250712  EPI_ISL_755631  EPI_ISL_456327  EPI_ISL_456301  EPI_ISL_456168  EPI_ISL_456193  EPI_ISL_548059  EPI_ISL_730010  EPI_ISL_825714  EPI_ISL_1013453  EPI_ISL_944786  EPI_ISL_954301  EPI_ISL_635116  EPI_ISL_493358  EPI_ISL_493351  EPI_ISL_666593  EPI_ISL_417444  EPI_ISL_548942  EPI_ISL_596543  EPI_ISL_873165  EPI_ISL_1225364  EPI_ISL_496772  EPI_ISL_693478  EPI_ISL_693479  EPI_ISL_693480  EPI_ISL_729891  EPI_ISL_1111176  EPI_ISL_536510  EPI_ISL_536489  EPI_ISL_1093165  EPI_ISL_833336  EPI_ISL_941340  EPI_ISL_1138775  EPI_ISL_960670  EPI_ISL_912388  EPI_ISL_815391  EPI_ISL_738343  EPI_ISL_807154  EPI_ISL_925905  EPI_ISL_751217  EPI_ISL_678072  EPI_ISL_678158  EPI_ISL_1167139  EPI_ISL_1167161  EPI_ISL_1167135  EPI_ISL_754100  EPI_ISL_875532  EPI_ISL_1063695  EPI_ISL_831245  EPI_ISL_517662  EPI_ISL_510868  EPI_ISL_934389  EPI_ISL_1199586  EPI_ISL_648160  EPI_ISL_594152  EPI_ISL_693840  EPI_ISL_796181  EPI_ISL_738065  EPI_ISL_534336  EPI_ISL_1010730  EPI_ISL_1074019  EPI_ISL_707707  EPI_ISL_717697  EPI_ISL_717700  EPI_ISL_1118884  EPI_ISL_707700  EPI_ISL_803120  EPI_ISL_1073806  EPI_ISL_1096069  EPI_ISL_954297  EPI_ISL_1112329  EPI_ISL_859932  EPI_ISL_407071  EPI_ISL_1009808  EPI_ISL_1017213  EPI_ISL_1159050  EPI_ISL_1171100  EPI_ISL_1220967  EPI_ISL_1234033  EPI_ISL_873201  EPI_ISL_876142  EPI_ISL_983856  EPI_ISL_979505  EPI_ISL_873262  EPI_ISL_527660  EPI_ISL_812342  EPI_ISL_812672  EPI_ISL_626344  EPI_ISL_626349  EPI_ISL_641501  EPI_ISL_641409  EPI_ISL_641514  EPI_ISL_683012  EPI_ISL_683020  EPI_ISL_683026  EPI_ISL_683036  EPI_ISL_683055  EPI_ISL_683069  EPI_ISL_641415  EPI_ISL_683079  EPI_ISL_683088  EPI_ISL_683098  EPI_ISL_683114  EPI_ISL_683126  EPI_ISL_683139  EPI_ISL_683147  EPI_ISL_683159  EPI_ISL_683169  EPI_ISL_683179  EPI_ISL_641428  EPI_ISL_683189  EPI_ISL_683193  EPI_ISL_641437  EPI_ISL_683208  EPI_ISL_683224  EPI_ISL_683246  EPI_ISL_683261  EPI_ISL_683269  EPI_ISL_683286  EPI_ISL_683294  EPI_ISL_683299  EPI_ISL_683310  EPI_ISL_683322  EPI_ISL_641453  EPI_ISL_641471  EPI_ISL_641479  EPI_ISL_641488  EPI_ISL_523012  EPI_ISL_523055  EPI_ISL_523058  EPI_ISL_577778  EPI_ISL_577780  EPI_ISL_577788  EPI_ISL_577807  EPI_ISL_577808  EPI_ISL_577810  EPI_ISL_577815  EPI_ISL_577819  EPI_ISL_577821  EPI_ISL_577822  EPI_ISL_577825  EPI_ISL_577828  EPI_ISL_632452  EPI_ISL_632380  EPI_ISL_632457  EPI_ISL_632458  EPI_ISL_632459  EPI_ISL_632436  EPI_ISL_632334  EPI_ISL_632489  EPI_ISL_722414  EPI_ISL_722416  EPI_ISL_722418  EPI_ISL_447628  EPI_ISL_447630  EPI_ISL_447633 |

**Table S4**. **Results of model selection of clade only individual and shared FLC models**. Estimates include the tMRCA of phylogeny, tMCRA of the Netherlands and Denmark clades, and the evolutionary rates for the whole phylogeny, and the Netherlands and Denmark clades. The 95% HPD interval is shown in brackets.

| Model | tMRCA | Netherlands tMRCA | Denmark tMRCA | Estimated evolutionary rate (mean) | Netherlands evolutionary rate | Denmark evolutionary rate |
| --- | --- | --- | --- | --- | --- | --- |
| FLC (clade) | **20-07-2019** [02-07-2019, 24-09-2019] | **14-03-2020** [17-02-2020, 25-03-2020] | **28-12-2019** [10-11-2019,13-02-2020]* | **4.75** $\times{10}^{-4}$  [4.37 $\times{10}^{-4}$,  5.16 $\times{10}^{-4}$]* | **6.06** $\times{10}^{-4}$ [4.12 $\times{10}^{-4}$, 8.23 $\times{10}^{-3}$] | **2.3** $\times{10}^{-4}$ [1.65 $\times{10}^{-4}$, 3 $\times{10}^{-4}$] |
| FLC (shared, clade) | **20-07-2019** [02-07-2019, 15-08-2019] | **06-03-2020** [17-02-2020, 25-03-202] | **24-02-2020** [01-01-2020, 08-04-2020] | **4.77** $\times{10}^{-4}$ [4.36 $\times{10}^{-4}$, 5.17$\times{10}^{-4}$]* | **6.33** $\times{10}^{-4}$ [4.28 $\times{10}^{-4}$, 8.45 $\times{10}^{-4}$] | |
| * ESS < 200 | | | | | | |

**Table S5.** **Estimates generated from FLC clock models with a gamma prior on the clock rate.** Estimates include the evolutionary rates (substitution/site/year) estimated for the whole phylogeny, and the Netherlands and Denmark clades. The 95% HPD interval is shown in brackets.

| Model | Estimated evolutionary rate (mean) | Netherlands evolutionary rate | Denmark evolutionary rate |
| --- | --- | --- | --- |
| FLC (clade*) | **4.56** $\times{10}^{-4}$  [4.14 $\times{10}^{-4}$, 4.97 $\times{10}^{-4}$]* | **6.1** $\times{10}^{-4}$ [4.04 $\times{10}^{-4}$, 8.17 $\times{10}^{-4}$] | **2.34** $\times{10}^{-4}$ [1.68 $\times{10}^{-4}$, 3.06 $\times{10}^{-4}$] |
| FLC (stem & clade*) | **4.64** $\times{10}^{-4}$ [4.26 $\times{10}^{-4}$, 5.06$\times{10}^{-4}$] | **1.83** $\times{10}^{-3}$ [1.3 $\times{10}^{-3}$, 2.41 $\times{10}^{-3}$] | **2.**$\times{10}^{-4}$ [1.76 $\times{10}^{-4}$, 3.17 $\times{10}^{-4}$] |
| FLC (shared, clade*) | **4.75** $\times{10}^{-4}$ [4.35 $\times{10}^{-4},$ 5.17 $\times{10}^{-4}$] | **6.32** $\times{10}^{-4}$ [4.21 $\times{10}^{-4}$, 8.41 $\times{10}^{-4}$] | |
| FLC (shared, stem & clade*) | **4.71** $\times{10}^{-4}$ [4.33 $\times{10}^{-4}$, 5.09 $\times{10}^{-4}$] | **1.83** $\times{10}^{-3}$ [1.29 $\times{10}^{-3}$, 2.4 $\times{10}^{-3}$] | |
| * ESS < 200 |  |  | |
